# Drug Discovery Opportunities at the Endothelin B Receptor-Related Orphan G Protein-Coupled Receptors, GPR37 and GPR37L1

**DOI:** 10.3389/fphar.2015.00275

**Published:** 2015-11-17

**Authors:** Nicola J. Smith

**Affiliations:** ^1^Molecular Cardiology Program, Victor Chang Cardiac Research Institute, Darlinghurst, NSW, Australia; ^2^St. Vincent’s Clinical School, University of New South Wales, Darlinghurst, NSW, Australia

**Keywords:** GPR37, GPR37L1, orphan, G protein-coupled receptor, Pael-R, parkin, endothelin, ET_B_

## Abstract

Orphan G protein-coupled receptors (GPCRs) represent a largely untapped resource for the treatment of a variety of diseases, despite sophisticated advances in drug discovery. Two promising orphan GPCRs are the endothelin B receptor-like proteins, GPR37 [ET(B)R-LP, Pael-R] and GPR37L1 [ET(B)R-LP-2]. Originally identified through searches for homologs of endothelin and bombesin receptors, neither GPR37 nor GPR37L1 were found to bind endothelins or related peptides. Instead, GPR37 was proposed to be activated by head activator (HA) and both GPR37 and GPR37L1 have been linked to the neuropeptides prosaposin and prosaptide, although these pairings are yet to be universally acknowledged. Both orphan GPCRs are widely expressed in the brain, where GPR37 has received the most attention for its link to Parkinson’s disease and parkinsonism, while GPR37L1 deletion leads to precocious cerebellar development and hypertension. In this review, the existing pharmacology and physiology of GPR37 and GPR37L1 is discussed and the potential therapeutic benefits of targeting these receptors are explored.

## Introduction

G protein-coupled receptors (GPCRs) are a family of seven transmembrane (TM)-spanning proteins that transmit responses from the extracellular milieu by binding to a variety of different ligands, including neurotransmitters, lipids, peptides, protons, odorants, and light. They are considered to be ideal drug targets because of their propensity to bind and respond to agonists, antagonists and allosteric modulators, facilitating a plethora of functional outcomes. The rhodopsin or Class A family of 296 GPCRs (Lagerstrom and Schioth, 2008) remains the largest source of therapeutic targets; as of 2014, 79 rhodopsin-like GPCRs were the target of FDA-approved drugs and a further 46 were part of ongoing clinical trials (Rask-Andersen et al., 2014). However, a large number of the rhodopsin-like receptors are considered to be “orphans” as they are yet to be paired with their cognate endogenous ligand. In this review, the discovery, description and possible therapeutic application of two endothelin B receptor-like peptide family GPCRs, GPR37 and GPR37L1, will be described.

## Endothelins and the Endothelin B Receptor

**FIGURE 1:**
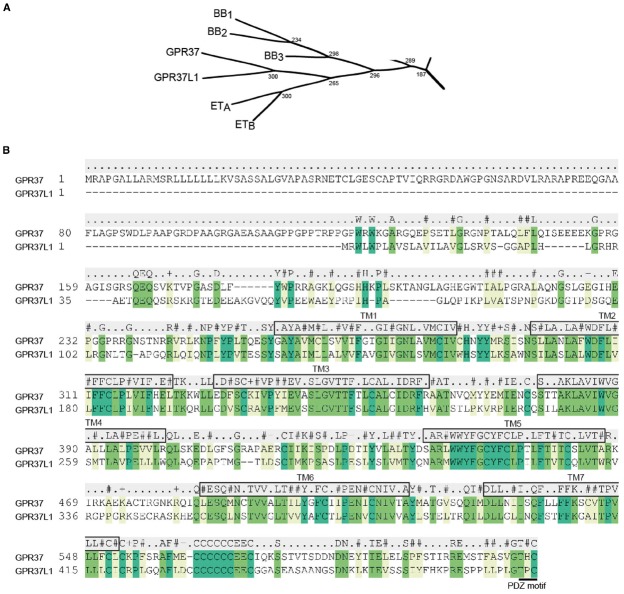
**GPR37, GPR37L1, and their closest relatives. (A)** Subsection of the β-subgroup of peptide receptors showing the phylogenetic relationship between GPR37 and GPR37L1 and the endothelin (ET_A_ and ET_B_) and bombesin (BB_1_, BB_2_, and BB_3_) receptors. Phylogenetic tree adapted from Fredriksson et al. (2003). **(B)** Alignment of human GPR37 and GPR37L1 highlighting sequence conservation in the TM regions. Residues with conserved identity are listed above the alignment, while conserved similarity is indicated for hydrophobic (#), positively charged (+), negatively charged (–), or unrelated (.) residues. Predicted TM domains are boxed and labeled and the C-terminal PDZ motif is underlined.

Endothelin-1 (ET-1) was first discovered in 1988 (Yanagisawa et al., 1988) and remains the most potent endogenous vasoconstrictor described to date. There are three endothelin isoforms, ET-1, ET-2 and ET-3, which are 21-amino acid peptides with two intra-chain disulfide bonds (Inoue et al., 1989). ET-1 and ET-2 are equipotent at both the ET_A_ (Arai et al., 1990) and the ET_B_ (Sakurai et al., 1990) receptors, while ET-3 is selective for ET_B_. The ET family is best known for its role in mediating potent and long acting vasoconstriction; ET-1 is released from the vascular endothelium and largely acts in a paracrine manner on smooth muscle ET_A_ receptors (Kohan et al., 2011). ET levels are controlled by the ET_B_ receptor, which acts as a “clearance” receptor on the lung endothelium by binding and internalizing circulating endothelins (Kohan et al., 2011; Mazzuca and Khalil, 2012). Therapeutic applications of ET antagonists include the treatment of pulmonary artery hypertension, where dual ET_A_/ET_B_ antagonists have been used in the clinic since 2002 (Rubin et al., 2002; Kohan et al., 2011; Mazzuca and Khalil, 2012), while the ET_B_ agonist IRL1620 may be useful for enhancing chemotherapeutic delivery and has recently entered phase II clinical trials (Maguire and Davenport, 2014). In the late 1990s, the cloning of GPR37 and GPR37L1 suggested that the endothelin family of receptors may have doubled in size (Figure 1A). However, neither receptor has been found to respond to endothelins or related peptides, nor have they been linked to vasoconstriction or pulmonary artery hypertension. The putative functions of these ET_B_-like receptors are outlined below.

## Discovery of GPR37 and GPR37L1

### Identification of GPR37

The orphan GPCR, GPR37, was first described in 1997 by Zeng et al. (1997), who discovered a 614 amino acid transcript through expressed sequence tag (EST) analysis of a human hippocampal library. The receptor was named human endothelin B receptor-like protein [hET(B)R-LP] after its closest homolog, the ET_B_ receptor, to which it shared 52% similarity and 26.7% identity. Analysis of the receptor sequence revealed four potential N-linked glycosylation sites, all within the unusually long N-terminus, at Asn^36^, Asn^138^, Asn^222^, and Asn^239^ (Zeng et al., 1997). At the same time, Marazziti et al. (1997) identified what they called GPR37 after sequencing random clones generated from a human frontal lobe library and searching for novel ESTs. They reported that this new gene mapped to chromosome 7q31, contained two exons with an intron that interrupted the sequence in TM3 and matched the bombesin 2 receptor (BB_2_), BB_1_ and ET_B_ with 45, 45, and 41% overall sequence homology, respectively (TM-based homology showed GPR37 shared the highest identity with the ET_B_ receptor at 46.4%). As with Zeng et al. (1997), they also noted the length of the N-terminus (234 amino acids) and identified a putative signal peptide (Marazziti et al., 1997). Subsequent studies also reported identification of GPR37 while searching for homologs of either the bombesin (Donohue et al., 1998), ET_B_ (Valdenaire et al., 1998), or both endothelin and bombesin families (Leng et al., 1999). The murine ortholog was localized to chromosome 6 and found to have 83% identity to human GPR37 and similar gene organization (Marazziti et al., 1998).

### Identification of GPR37L1

A year after the discovery of GPR37, Valdenaire et al. (1998) identified both GPR37 and a very highly related novel transcript encoding what they called ET(B)R-LP-2 (later termed GPR37L1), as part of a screen for ET_B_ receptor homologs (Figure 1B). GPR37L1 was identified as a faint band from a human caudate nucleus library screen that was then purified, sequenced and used to re-screen the library. The complete clone had an open reading frame of 1443 base pairs and encoded a 481 amino acid GPCR with 68% similarity and 48% identity to GPR37. Sequence analysis revealed only one potential glycosylation site, Asn^105^, in the N-terminus and a putative protein kinase C phosphorylation site at Tyr^332^ at the beginning of ICL3. Alignment of GPR37 and GPR37L1 revealed the presence of a cysteine cluster at the end of what is now recognized as helix 8, which they postulated could be a site for palmitoylation (Valdenaire et al., 1998). Subsequently, both GPR37 and GPR37L1 orthologs were cloned from a rat hypothalamic library: a 603 amino acid 7TM receptor homologous to GPR37, named GPCR/CNS1, and a 481 amino acid GPR37L1, originally named GPCR/CNS2 (Donohue et al., 1998). The rat receptors shared 42% homology and were highly similar to the human versions identified by both Valdenaire et al. (1998) and Donohue et al. (1998).

### Expression Patterns of GPR37 and GPR37L1

Both GPR37 and GPR37L1 are very highly expressed in the central nervous system, with little peripheral expression in humans and rodents. Northern blot analyses by several groups universally demonstrated that GPR37 is present in the brain, particularly the corpus callosum and substantia nigra (Marazziti et al., 1997; Zeng et al., 1997; Donohue et al., 1998), with some expression in the spinal cord, placenta, liver, stomach, and testis (Marazziti et al., 1997, 1998; Zeng et al., 1997; Donohue et al., 1998; Valdenaire et al., 1998; Leng et al., 1999). Reports of expression in other peripheral tissues varied, with Marazziti et al. (1997) failing to detect GPR37 transcript in human kidney, skeletal muscle, heart, lung or pancreas, while Leng et al. (1999) reported low levels in rat heart, liver, kidney and lung, and Zeng et al. (1997) detected a very faint band by Northern blotting in both the human kidney and pancreas. Within the brain, GPR37 is localized to neurons including the Purkinje cells of the cerebellum, pyramidal cells of the hippocampus and granule cells of the dentate gyrus, as confirmed by in-situ hybridization (Zeng et al., 1997). To date, the physiological significance of GPR37 has been largely related to its expression in the brain.

In contrast to the predominantly neuronal distribution of GPR37, GPR37L1 is expressed exclusively in glial cells within the brain, with in-situ hybridization revealing greatest intensity of GPR37L1 within the Bergmann glia of the cerebellum (Valdenaire et al., 1998). According to Northern blot analysis in human tissue, GPR37L1 is highly enriched in the cerebellum and throughout the brain, with no detectable expression in the periphery, including heart, kidneys, placenta or pancreas (Valdenaire et al., 1998). This contrasts with rodent Northern blots, where heart, pituitary, stomach, lung and some liver and kidney transcripts were identified (Leng et al., 1999). More recent studies, however, have identified peripheral GPR37L1 expression by either qPCR or Western blot analysis in human heart explants (Min et al., 2010) and the gastrointestinal system (Ito et al., 2009), respectively. Thus, while it is likely that the majority of the physiological effects of GPR37L1 are linked to its localization in the central nervous system, it is possible that GPR37L1 also plays a role in the periphery.

## Pharmacology and Biochemistry of GPR37 and GPR37L1

### Potential Pharmacology of GPR37 and GPR37L1

Because of their relative homology to the endothelin and bombesin receptors, initial efforts to define the pharmacology of GPR37 and GPR37L1 focused on testing ligands from these related receptors in radioligand binding and signaling assays. For example, GPR37 has been screened for binding of [^125^I]ET-1 and [^125^I] ET-3 (Marazziti et al., 1997; Zeng et al., 1997; Valdenaire et al., 1998), and [^125^I]bombesin (Marazziti et al., 1997) without demonstrable specific binding. Heterologous expression of GPR37 in *Xenopus* oocytes or HEK293 cells and stimulation with bombesin, gastrin-releasing peptide, neuromedin B, Bim 26292, ET-1, ET-2, or ET-3 (Leng et al., 1999) or ET-1, ET-3, bombesin and NPY (Zeng et al., 1997) failed to generate calcium currents, or calcium and cAMP signaling, respectively. Similar experiments were performed at GPR37L1 with identical results. Valdenaire et al. (1998), for example, stably expressed GPR37L1 in HEK293 cells and assessed binding to radiolabeled ET-1 and ET-3, bombesin, CCK-8 and gastrin-releasing peptide, while Donohue et al. (1998) microinjected GPR37L1 into Xenopus oocytes or transfected BALB/B1 fibroblasts and examined [^125^I]-Bn and [^125^I]-[DTyr^6^,βAla^11^,Phe^13^,Nle^14^]Bn-(6-14) binding and agonism (calcium and inositol phosphates). Thus, while both GPR37 and GPR37L1 are closely related to the endothelin and bombesin receptor families, they are unable to bind to the cognate ligands for these receptors.

### Head Activator and Prosaposin: Deorphanization of GPR37 and GPR37L1?

The first ligand proposed to be the endogenous partner of GPR37 was HA, an undecapeptide (pGlu-Pro-Pro-Gly-Gly-Ser-Lys-Val-Ile-Leu-Phe) originally discovered in *Hydra* and reported to have a human homolog (Bodenmuller and Schaller, 1981; Rezgaoui et al., 2006). The authors found that 2 nM treatment of GPR37 transiently- or stably-transfected cells led to receptor internalization and FRET-based co-localization of HA and GPR37, although the images presented were not entirely consistent with conventional patterns of GPCR internalization. Intriguingly, when HA-mediated calcium stimulation was measured using a Gα_16_/aequorin assay, GPR37 expression led to translation of HA concentration-response curves along the Y-axis without a change in potency. This unusual pharmacology was attributed to endogenous GPR37 already present in the cells, as detected by Western blot, yet the authors note that they failed to detect GPR37 transcript by Northern blot (Rezgaoui et al., 2006), which would ordinarily suggest that the antibody used for blotting was not specific. Similarly, HA was reported by Gandia et al. (2013) to stimulate GPR37 internalization, calcium-mediated nuclear factor of activated T-cells reporter gene transcription and inhibition of cAMP accumulation. Interestingly, calcium and cAMP responses were also augmented (again with an apparent translation along the Y-axis) in a GPR37 deletion mutant, GPR37^Δ563–568^, which lacked a 6-Cysteine motif in the C-terminus shown to contribute to the intracellular retention of GPR37, although the concentration-response curves shown did not include concentrations at which HA had no effect on signal transduction, complicating the interpretation of these findings (Gandia et al., 2013). These studies contrast with that of Dunham et al. (2009), who attempted to replicate the finding that HA was a ligand for GPR37 but found no evidence of HA-mediated internalization, ERK1/2 phosphorylation or cAMP stimulation. HA was also included in a larger screen of all remaining orphan GPCRs but did not register as a “hit” for any GPCR tested (Southern et al., 2013). Finally, perhaps the most damning evidence against HA as the endogenous ligand for GPR37 is the fact that it has not been found in the human genome (Davenport et al., 2013). Thus, it appears that HA is unlikely to be an agonist at GPR37 and is certainly not its endogenous ligand.

More recently, GPR37 and GPR37L1 were simultaneously paired with the endogenous protein prosaposin and its active peptide fragment, prosaptide (the synthetic analog is called TX14A; Meyer et al., 2013). In this study, a series of known neuropeptides was screened against GPR37 and GPR37L1 and TX14A was found to induce internalization of both receptors. Biotinylated TX14A was able to immunoprecipitate both GPR37 and GPR37L1, but not their closest relative, the ET_B_ receptor, or other controls. Based upon ERK1/2 phosphorylation, [^35^S]-GTPγS accumulation and inhibition of forskolin-stimulated cAMP in HEK293T cells co-expressing each receptor, it was concluded that GPR37 and GPR37L1 were Gα_i_-coupled receptors, consistent with the previously reported role of both TX14A and prosaposin in the brain (Hiraiwa et al., 1997; Campana et al., 1998; Misasi et al., 1998). To confirm activity against the endogenous receptors, the authors turned to primary cortical astrocytes and down-regulated the expression of the receptors using siRNA. TX14A was shown to induce ERK1/2 phosphorylation in a GPR37-dependent manner, while either GPR37 or GPR37L1 deletion was sufficient to prevent TX14A-mediated neuroprotection. Because the endogenous source of TX14A is the neuroprotective protein, prosaposin, the experiments were repeated using recombinant prosaposin and the same effects were observed (Meyer et al., 2013). Thus, the authors concluded that prosaposin is the endogenous ligand for both GPR37 and GPR37L1.

Again, the claim that prosaposin and prosaptide are endogenous ligands for GPR37 and GPR37L1 is not without some controversy and is yet to be ratified by the International Union of Basic and Clinical Pharmacology (IUPHAR) Nomenclature Committee. First, the concentration of prosaposin or TX14A required for agonism is 100 nM, much higher than traditionally associated with peptide/GPCR interactions (e.g., ET-1 binds to the ET_A_ and ET_B_ receptors with p*K*d 9.1–10.5 and 9.2–10.0 affinity, respectively; Alexander et al., 2013). Second, Gα_i/o_-mediated agonism via [^35^S]-GTPγS led to only a very small increase in signal above baseline (approximately 8 and 6% above unstimulated levels for GPR37 and GPR37L1, respectively), in stark contrast to the doubling usually seen for Gα_i/o_-coupled GPCRs in this assay (e.g., methacholine stimulates a 95% increase above baseline at the M_2_ muscarinic receptor; Mistry et al., 2011), while both propionate and 4-CMTB stimulate >100% Gα_i/o_-[^35^S]-GTPγS incorporation via the free fatty acid 2 receptor (Smith et al., 2011). Sub-optimal conditions for the [^35^S]-GTPγS assay, which is sensitive to concentration of GDP in a receptor-dependent manner (Mistry et al., 2011), are unlikely to explain the small signal window as TX14A-mediated cAMP inhibition was also only 10–15%. An alternative possibility is that both receptors are highly constitutively active or that there is saturating endogenous prosaposin already present in the media, leading to the assay maximum already being reached; normalization of the data precluded assessment of such activity from the presented figures. Third, like HA, TX14A was also included in the MRC Technologies β-arrestin-based orphan GPCR screen but was not detected as a ligand at either GPR37 or GPR37L1 (Southern et al., 2013), although it should be noted that Meyer et al. (2013) did not specifically investigate β-arrestin signaling with these ligands. Finally, in the siRNA knockdown experiments using primary cortical astrocytes, it is unclear how GPR37L1 siRNA was able to block neuroprotection by TX14A or prosaposin as Western blot analysis of whole brain and primary astrocytes suggest that the receptor is markedly downregulated in culture (Meyer et al., 2013). Thus, a number of questions remain regarding the pairing of prosaposin with GPR37 and GPR37L1.

While direct agonism remains to be independently demonstrated for either receptor, it seems likely that prosaposin and TX14A have some influence on GPR37 subcellular localization, although the evidence itself is contradictory. For example, Lundius et al. (2014) created stable GPR37-turboGFP-expressing catecholaminergic N2a cells and demonstrated by fluorescence correlation spectroscopy that GPR37 is co-localized with TX14A and GM1 gangliosides in lipid rafts. Interestingly, immunoabsorption of extracellular prosaposin reduced GPR37 cell-surface expression (Lundius et al., 2014), an effect that is opposite to what would be predicted for depletion of an agonist (receptor upregulation) and in contrast to the ligand-mediated internalization first reported (Meyer et al., 2013). Further study is clearly required to understand the complex interplay between prosaposin and GPR37.

### Cell Surface Expression, Cytotoxicity, and a PDZ Motif at the C-Terminus of GPR37

Since it was first discovered, an ongoing issue with studying GPR37 has been its poor cell surface expression and cytotoxicity in heterologous expression systems. For example, C-terminally tagged GPR37 transient or stable expression in COS-7 and HEK293 cells, respectively, resulted in predominantly intracellular localization of the receptor (Zeng et al., 1997) and associated cell death (Imai et al., 2001). Because GPR37 has been linked to Parkinson’s disease (*see below*), efforts have focused on enhancing GPR37 cell surface levels so that the receptor is amenable to study. One approach has been to rescue expression through the use of a chemical chaperone, 4-phenylbutyrate (4-PBA; Kubota et al., 2006). Known to restore cell surface expression of the cystic fibrosis TM conductance regulator (CFTR) mutant, ΔF508, 4-PBA was shown to increase cell surface staining and reduce the concomitant cytotoxicity that resulted from stable integration of GPR37 in SH-SY5Y neuroblastoma cells (Kubota et al., 2006). An alternative approach was adopted by Randy Hall’s group, who hypothesized that cell surface expression could be rescued by truncation of the long, unstructured GPR37 N-terminus (Dunham et al., 2009), as had been shown for other GPCRs (Dunham and Hall, 2009). Indeed, cell surface expression was restored by deletion of the entire N-terminus, as measured by luminometry and flow cytometry against an N-terminal FLAG tag, with at least the first 210 residues requiring deletion for rescue to occur (Dunham et al., 2009). More recently, Gandia et al. (2013) demonstrated that the region responsible for intracellular retention of GPR37 was a 6-Cysteine motif in the C-terminus (Cys^563^–Cys^568^) by generating multiple truncation or deletion mutants. An alternative strategy to genetic manipulation for increasing cell surface expression of GPCRs is heterodimerization (Dunham and Hall, 2009; Smith and Milligan, 2010), which was employed by Dunham et al. (2009), who found that both the dopamine D_2_ and adenosine A_2A_ receptors enhanced GPR37 trafficking. Furthermore, co-expression of GPR37 and D_2_ was associated with enhanced D_2_ ligand affinity in [^3^H]-spiperone competition binding assays (the Δ210 GPR37 construct was used because of its more favorable expression profile), although this altered affinity did not translate into changes in D_2_-Gα_o_ fusion protein [^35^S]-GTPγS signaling nor D_2_ receptor trafficking (Dunham et al., 2009).

GPR37 and GPR37L1 both contain PDZ binding motifs at the end of their C-terminus, raising the possibility that cell surface expression could be enhanced by direct interactions at this site. Thus, Hall’s team also probed for novel binding partners of GPR37 by using its PDZ domain-containing C-terminus as bait, which led to the discovery that GPR37, and later GPR37L1 (Dutta et al., 2014), interact with the atypical PDZ scaffold protein, syntenin-1 (Dunham et al., 2009). A subsequent yeast 2-hybrid screen by Dutta et al. (2014) found that both GPR37 and GPR37L1 interact with the scaffold/adaptor protein PICK1 (protein interacting with C-kinase) and that this interaction is specific to the C-terminal PDZ motif in GPR37 and the PDZ domain in PICK1. Both receptors also bound to GRIP4/5 (Dutta et al., 2014). In contrast to the effects on GPR37 expression observed with syntenin-1 (Dunham et al., 2009), co-expression of PICK1 and GPR37 led to a reduction in GPR37 expression levels and a concomitant reduction in GPR37-mediated cell death (Dutta et al., 2014). Finally, GPR37 has been shown to interact specifically with multi-PDZ domain protein 1 (MUPP1) via its eleventh PDZ domain to form a scaffold with contactin-associated protein-like 2 (CASPR2), which has been linked to autism spectrum disorders (*see later*; Tanabe et al., 2015). Using mouse brain homogenates, the authors demonstrated that a GST-CASPR2 C-terminal fusion protein pulled-down both MUPP1 and GPR37 (as well as GABA_B2_), while the GST-GPR37 C-terminus, but not a GST-GPR37 fusion lacking its PDZ domain, was able to pull down both MUPP1 and CASPR2 (Tanabe et al., 2015). The physiological significance of any of these PDZ motif-mediated interactions remains to be determined, although there is some evidence that disruption of the GPR37-MUPP1-CASPR2 interaction may be linked to autism spectrum disorders (*see later*).

## Potential Roles of GPR37 in Health and Disease

### GPR37 Neurotoxicity and Parkinson’s Disease

The cytotoxicity seen upon heterologous expression of GPR37 actually provides a clue to a key pathophysiological role of GPR37. In a seminal 2001 paper by Imai et al. (2001) in Cell, GPR37 was isolated from a human brain library in a yeast 2-hybrid screen for novel interacting partners of parkin, and thus named “parkin-associated endothelin receptor-like receptor” (Pael-R). Parkin is of great therapeutic interest because mutations in this gene are directly linked to autosomal recessive juvenile parkinsonism (ARJP), a form of parkinsonism notable for its absence of Lewy bodies. Critically, the authors found that insoluble (and therefore misfolded) GPR37 was increased in patients with AR-JP, linking the aggregation of GPR37 to disease pathogenesis (Imai et al., 2001). GPR37 aggregates were later also found in a variety of inclusion bodies in a study by Murakami et al. (2004), who probed the brains from patients with Parkinson’s disease (PD, *n* = 6), dementia with Lewy bodies (DLB, *n* = 3), multiple system atrophy (MSA, characterized by glial cytoplasmic inclusions, *n* = 6) and six control subjects. In this study, GPR37 aggregates were identified within the Lewy bodies of PD brain sections, residing predominantly in the core and sometimes the halo region of the inclusion. DLB tissue also stained positive for GPR37 in the same pattern, with GPR37 staining coinciding with parkin and ubiquitin immunoreactivity in both PD and DLB (Murakami et al., 2004). No GPR37 was detected in patients with MSA. Thus, GPR37 aggregation appears to be a hallmark of parkinsonism (AR-JP and PD) and other, although not all, inclusion-related neurological diseases.

#### Molecular Mechanism of GPR37-Mediated Neurotoxicity

**FIGURE 2:**
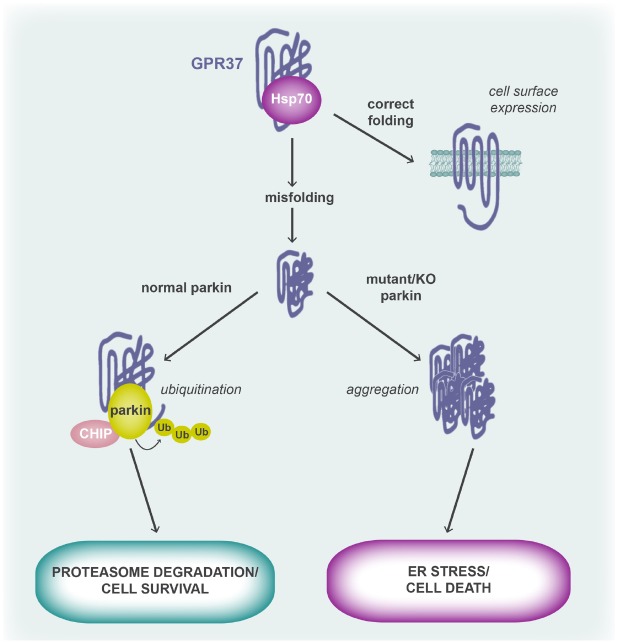
**GPR37 (mis)folding and its role in cell fate.** Folding of the GPR37 polypeptide chain is aided by the molecular chaperone, Hsp70. When the receptor is correctly folded, it is trafficked to the cell surface where it is able to respond to agonist. However, when the receptor is misfolded, parkin and CHIP displace Hsp70 from the GPR37 polypeptide and parkin, an E3 ubiquitin ligase, polyubiquitinates GPR37 to target it for proteasomal degradation. In cases where parkin is mutated, such as in AR-JP, or in parkin KO mice, misfolded GPR37 cannot be cleared from the cell and thus forms aggregates. This leads to activation of the unfolded protein response and ER stress, causing cell death. It is thought that GPR37 protein aggregation contributes to the DA neurotoxicity seen in AR-JP patients who have mutations in parkin.

Parkin is an E3 ubiquitin-protein ligase that was itself first identified in a genetic study of patients with AR-JP, where it was found to be highly expressed in the brain, particularly the substantia nigra (Kitada et al., 1998). Mutations in parkin that have been associated with AR-JP specifically enhance dopaminergic (DA) neuronal cytotoxicity by failing to appropriately remove aggregated proteins via the proteasome, causing the protein aggregates to trigger the unfolded protein response (UPR) to cause cell death (Figure 2). It is specifically through the loss of E3 ubiquitin ligase activity that parkin mutations are pathogenic (Imai et al., 2000; Shimura et al., 2000; Zhang et al., 2000), leading to the accumulation of a number of substrates including GPR37 (Imai et al., 2001). Under normal conditions, misfolded GPR37 is bound to the molecular chaperone, Hsc/Hsp70, which facilitates appropriate refolding (Imai et al., 2001, 2002). However, during ER stress, both parkin and another protein, CHIP (carboxyl terminus of the Hsc70-interacting protein), become upregulated and CHIP then promotes the dissociation of Hsc/Hsp70 from GPR37 (Imai et al., 2002). In the absence of Hsc/Hsp70, GPR37 interacts with parkin via its C-terminus and is subjected to poly-ubiquitination and proteasomal degradation, resulting in cytoprotection of the cell (Imai et al., 2001). In contrast, when parkin is unable to meet increased demand or has been mutated, unfolded GPR37 is not ubiquitinated and instead triggers the UPR and neuronal death, a process that can be rescued by re-expression or overexpression of wild type parkin (Imai et al., 2001; Figure 2). The possibility that GPR37 misfolding induces macroautophagy was more recently proposed, although the evidence was largely circumstantial (Marazziti et al., 2009). Finally, GPR37 has also been reported to interact with another E3 ubiquitinase, HDR1, to the same end (Omura et al., 2006). GPR37L1 does not undergo ubiquitination (Imai et al., 2001) and thus the phenomenon is limited to GPR37.

#### GPR37 Knockout Mice and Dopaminergic Neurotoxicity

A largely unresolved issue in the relationship between GPR37 and neurotoxicity is that GPR37 misfolding seems to specifically contribute to the loss of DA neurons, despite its broader expression pattern that encompasses non-DA neurons and oligodendrocytes (Chung et al., 2001). For example, overexpression of GPR37 in transgenic *Drosophila* resulted in an age-dependent degradation of DA neurons, regardless of whether the promoter used was pan-neuronal or DA-specific (Yang et al., 2003). Meanwhile, despite displaying normal neuroanatomy and largely unaltered components of the DA signaling pathway, global GPR37 knockout (KO) mice have been shown to display subtle alterations to DA signaling (Marazziti et al., 2004, 2007, 2011). In a series of different studies with the same mice (with increasing number of backcrosses over the years), Marazziti et al. (2004) have found GPR37 KO mice to have significantly decreased striatal DA and elevated dopamine transporter (DAT) activity due to increased presynaptic plasma membrane expression in the striatum (Marazziti et al., 2007; the same mice have also been shown to display increased anxiety, discussed below in Section “Major Depressive Disorder, Bipolar Disorder, and Non-Motor Attributes of Parkinsonism”; Mandillo et al., 2013). Furthermore, the increased DAT surface trafficking and activity was shown to be due to loss of intracellular retention by GPR37 binding (Marazziti et al., 2007), although a separate study failed to see a specific interaction between GPR37 and DAT (Dunham et al., 2009). Consistent with the finding that overexpression of GPR37 enhanced the affinity of various ligands at D_2_ receptors (Dunham et al., 2009), D_2_ receptor ligands had reduced affinity in GPR37 KO mice (Marazziti et al., 2007). Thus, while elements of the nigrostriatal DA pathway appear to be perturbed, the overall mechanism of these defects remains to be fully understood.

Several behavioral models have been used to explore the consequences of these subtle changes in DA signaling, with somewhat contradictory results. An early study found that GPR37 KO mice had significantly reduced locomotor activity in the open field test of general locomotion and exploratory behavior (Marazziti et al., 2004). These GPR37 KO mice were more sensitive to amphetamine administration than wild type littermates, indicated by increased activity, but displayed reduced motor co-ordination in the rotarod test of balance and agility. The authors noted that although GPR37 KO mice had significantly lower body weights than their littermates, this was not found to affect the results (Marazziti et al., 2004). In a subsequent study, Marazziti et al. (2007) found that GPR37 KO mice actually had *reduced* cocaine-stimulated locomotion; this is surprising as cocaine and amphetamine both act independently to increase acute extracellular DA concentrations (amphetamine causes release of DA from nerve terminals while cocaine prevents DA reuptake). The mice also had altered responsiveness to induction of catalepsy by either D_1_ or D_2_ antagonism, although no dose-dependence was observed (Marazziti et al., 2007). While locomotion and both Akt and ERK1/2 striatal signatures were differentially affected by cocaine and amphetamine, GPR37 KO mice failed to respond to either stimulus when used as an incentive in the conditional place preference behavioral test and did not develop behavioral sensitization over time (Marazziti et al., 2011). Finally, when measuring pilocarpine-induced tremulous jaw movement, a mouse model of PD tremor, GPR37 KO mice had significantly reduced tremulous jaw movements when compared to wild type mice, suggesting that a GPR37 antagonist could be an additional therapeutic approach for the treatment of classical PD tremor (Gandia et al., 2015). It should be noted, however, that the extent of DA involvement in PD tremor is unclear (Hallett, 2012). Thus, taken together, the behavioral effects of GPR37 KO resulting from DA pathway disruption remain to be fully characterized in the context of PD.

#### Interaction Between Parkin and GPR37 in Models of Neurotoxicity

One of the conundrums in the PD field is the mechanistic link between parkin, AR-JP and PD more generally. While mutations within parkin clearly cause AR-JP via DA neurotoxicity and nigrostriatal neuronal loss, how a recessive mutation in parkin can cause such a profound phenotype is unclear. Furthermore, deletion of parkin in mice does not recapitulate the symptoms seen in AR-JP and instead results in generally healthy animals that do not display nigrostriatal neurodegeneration over time, although they do show elevated DA levels and some behavioral deficits (Goldberg et al., 2003; Itier et al., 2003). For parkin mice to develop AR-JP-like symptoms, a “second hit” appears necessary, whether it be an additional genetic modification or chemical treatment [such as used in the 1-methyl-4-phenyl-1,2,3,6-tetrahydropyridine (MPTP) model of neurotoxicity]. Indeed, the spontaneous *quaking viable* mutant mouse that displays abnormal motor activity, tremor, quaking and seizures, is the result of simultaneous deletion of both parkin and the upstream parkin co-regulated gene (PACRG; Lockhart et al., 2004). Several studies have combined parkin deletion with changes to GPR37 expression in an attempt to recapitulate AR-JP. For example, adenoviral-mediated overexpression of GPR37 in the substantia nigra pars compacta led to neurotoxicity that was exacerbated in parkin KO mice (Kitao et al., 2007). This phenotype could be rescued by overexpression of the ER protein-folding chaperone, Orp150, or by treatment with the DA synthetase inhibitor, AMPT (a tyrosine hydroxylase blocker), thus linking GPR37 to ER stress and subsequent DA neuron death (Kitao et al., 2007). Similar results were observed in GPR37 transgenic mice crossed with parkin KO mice, which mimic AR-JP by progressively losing catecholaminergic neurons without the appearance of Lewy bodies (Wang et al., 2008). Tyrosine hydroxylase, DAT and vesicular monoamine transporter 2 were all downregulated in the striatum of these mice, consistent with the loss of DA nerve termini, while the UPR was chronically activated. Interestingly, despite the AR-JP-like neurodegenerative phenotype, no overt behavioral effects were observed in either the open field test or rotarod (Wang et al., 2008).

#### GPR37: Neuroprotective or Neurotoxic?

Excess levels of aggregated GPR37 are clearly neurotoxic while the proposed ligands for GPR37 are known to be neuroprotective, suggestive of a functional mismatch that is yet to be resolved. While the neurotoxicity seen upon GPR37 misfolding can be explained by failure to clear aggregated protein from the ER, it is harder to reconcile the survival benefits of GPR37 deletion with agonist-mediated neuroprotection via prosaposin. For example, GPR37 KO mice are actually resistant to the development of DA neurodegeneration and a PD-like phenotype in the MPTP model of PD neurotoxicity (Marazziti et al., 2004). Meanwhile, *in vitro* and *in vivo* treatment with a *retro-inverso* version of the prosaposin peptide, prosaptide D5, also protects DA neurons from the effects of MPTP administration (Liu et al., 2001). How overexpression and activation of the same receptor achieves the opposite phenotypic outcome is unknown, but may be related to the folding of GPR37. In a recent study, Lundius et al. (2013) examined the effect of GPR37-GFP overexpression on cytotoxicity mediated by three cytotoxic treatments that mimic components of PD: MPTP (a precursor to MPP+, an inhibitor of complex I that leads to mitochondrial dysfunction), rotenone (also causes mitochondrial dysfunction, leads to Lewy body-like aggregates) and 6-OHDA (forms reactive oxygen species and activates the UPR). Overall, GPR37 overexpression was neuroprotective and this was directly related to its increased cell surface expression (Lundius et al., 2013). Thus, it is possible that prosaposin acts as a chaperone for GPR37 to ensure correct folding and trafficking of the receptor, and that once at the cell surface, GPR37 signaling is itself neuroprotective. However, this theory is untested and cannot explain the neuroprotection, rather than absence of neurotoxicity, seen when GPR37 is deleted. Another possibility is that prosaposin’s neuroprotective effects are entirely independent of GPR37—an obvious experiment would be to assess the ability of prosaposin and prosaptide to enhance cell survival in GPR37 KO mice.

### Major Depressive Disorder, Bipolar Disorder, and Non-Motor Attributes of Parkinsonism

Tomita et al. (2013) identified GPR37 as a differentially expressed gene in the brains of major depressive disorder (*n* = 9) and bipolar disorder patients (*n* = 6) when compared to controls (*n* = 7). Samples of mRNA were taken for microarray analysis from the dorsolateral prefrontal cortex and anterior cingulate, regions of the brain linked to hedonism, impulse control, memory, learning, and depression. Their global analysis revealed that genes linked to cAMP signaling were increased in bipolar disorder but decreased in major depressive disorder (Tomita et al., 2013). Furthermore, the transcript levels of two orphan GPCRs, GPR37 and GPRC5B, matched these expression patterns in each disease. Dysregulation of GPR37 was confirmed by both qPCR and in-situ hybridization using cortical slices from a separate cohort of major depressive disorder and bipolar disorder patients. In contrast, a more recent transcriptome study failed to detect any GPR37 expression in the post-mortem brains of bipolar disordered patients (Cruceanu et al., 2015). Thus, while GPR37 may be downregulated in major depressive disorder, more studies are required to both confirm this result and determine the effect of bipolar disorder on GPR37 expression.

The potential interaction between GPR37 expression and affective disorders is also relevant to PD, as PD patients often experience non-motor symptoms involving mood, sleep, memory and learning and sensorimotor disturbances, as well as olfactory and gastrointestinal perturbations (Chaudhuri et al., 2006). To this end, Mandillo et al. (2013) used their previously described GPR37 KO mice to investigate non-motor behavioral effects of receptor deletion. Gender differences were also explored because although males have a higher prevalence of PD, females experience greater distress from the non-motor symptoms of PD (Scott et al., 2000; Negre-Pages et al., 2010). By comparing adult (4–6 months old) or aged (16–18 months old) wild type and GPR37 KO mice of both genders, the authors found a series of either gender-specific or genotype-specific differences, although these were generally all minor changes. For example, aged female GPR37 KO mice had significantly increased anxiety and depression-like behaviors, while olfactory function was marginally improved (Mandillo et al., 2013). Adult female mice also showed mild change in gastrointestinal function (increased stool frequency, reduced solid matter and increased water content), consistent with the noted expression of the receptor in the gut (Ito et al., 2009) and also with the phenotypes of DAT (Walker et al., 2000) and D_2_ receptor (Li et al., 2006) KO mice. Both male and female mice had reduced acoustic startle responses but no significant difference in prepulse inhibition (Mandillo et al., 2013). The results of this study support the idea that GPR37 may be responsible for some of the non-motor symptoms observed in PD, however a more recent study has found contradictory effects of GPR37 deletion.

Very recently, Lopes et al. (2015) carefully mapped GPR37 expression in the hippocampus and examined the effects of GPR37 deletion on hippocampal-related behaviors. GPR37 expression was localized predominantly in the extrasynaptic plasma membrane of dendritic spines and dendritic shafts, with the remainder largely localized intracellularly (Lopes et al., 2015), in agreement with Tanabe et al. (2015). While GPR37 deletion had no effect on either short- or long-term plasticity, the authors examined the effect of adenosine A_2A_ receptor blockade because that receptor is known to modulate synaptic plasticity and GPR37 cell surface expression is affected by co-expression with A_2A_ (Dunham et al., 2009). In general, there was little difference between untreated mice and those that received the A_2A_ antagonist, SCH58261, except for depotentiation after long-term potentiation, where depotentiation was delayed by SCH58261 in GPR37 KO but not wild type mice (Lopes et al., 2015). A_2A_ antagonism also specifically modified the response of GPR37 KO mice in the novel object recognition test of hippocampal-related working/reference memory. In both instances, the mechanism for these differences could not be explained. In the earlier study by Mandillo et al. (2013), older female mice were reported to have increased anxiety if they lacked GPR37. However, when Lopes et al. (2015) tested anxiety with either the marble burying test or elevated plus maze, they found that GPR37 KO mice both buried less marbles and spent more time in the open arm of the maze, respectively, indicating that they were actually less anxious than their wild type littermates. In this instance, treatment with the A_2A_ receptor antagonist had no effect on wild type anxiety but reverted the GPR37 KO responses back to that seen for wild type, i.e., the GPR37 KO mice became more anxious (Lopes et al., 2015). This too is a puzzling observation, as A_2A_ KO mice have been reported to show an anxiolytic, not anxiogenic, phenotype (Wei et al., 2014). Once again, the behavioral results are contradictory across GPR37 KO mice models. At the very least, we can conclude that GPR37 has no effect on working memory but that it may play a role in anxiety. Discovery of potent GPR37-specific ligands would undoubtedly help in this regard.

### Autism Spectrum Disorders

The gene for GPR37 is found within a locus called AUTS1, the first region of the human genome to be linked to autism spectrum disorder (ASD). Several SNPs have been identified within the coding region of GPR37, with two SNPs segregating with disease: a 1585–1587 TTC deletion (Del312F, in TM2) found in a single Japanese patient, and G2324A (R558Q, C-terminal) from a single Caucasian patient (Fujita-Jimbo et al., 2012). However, both of these mutations were also detected in an unaffected relative, suggesting that if either SNP is causal, it must only be so in the presence of a (presently unidentified) modifying gene mutation. The authors also noted that a T589M mutation in the GPR37 C terminus was present in seven affected men from five different Caucasian families, with only one carrier identified in the control cohort (Fujita-Jimbo et al., 2012). Each of the GPR37 mutations were overexpressed in C2C5 cells, where it was found that both GPR37 Del312F and GPR37 R558Q SNPs were more highly expressed than wild type GPR37 and that these cells had altered morphology (Fujita-Jimbo et al., 2012). In a very recent study, the authors further investigated the effects of the GPR37 R558Q SNP on the interaction between GPR37 and PDZ binding proteins, MUPP1 and CASPR2 (*see earlier*; Tanabe et al., 2015). Interestingly, in GST-CASPR2 C-terminal pull-down assays, GPR37 R558Q was less avidly bound to MUPP1 compared to WT GPR37 (only 14% of total GFP-MUPP1). Furthermore, GPR37 R558Q did not reach the cell surface and was not co-localized with MUPP1; while co-expression of wild type GPR37 with MUPP1 did not change receptor distribution, both were found at the cell surface (Tanabe et al., 2015). Finally, both MUPP1 and GPR37 were found to co-localize in the dendrites of hippocampal neuronal cell cultures, as previously described for GPR37 (Lopes et al., 2015). In contrast, GPR37 R558Q did not traffic to dendrites and although the number of branching primary dendrites was unaltered between wild type GPR37 and the GPR37 R558Q-expressing cells, dendrite length was significantly reduced (Tanabe et al., 2015). Further links between GPR37 and CASPR2, which has itself been linked to ASD (Bakkaloglu et al., 2008), were not examined and further study is clearly required to understand whether GPR37 mutations contribute to the complex phenotype of ASD.

### Testis Development

GPR37 expression was noted to be high in the testes in initial GPR37 cloning studies (Donohue et al., 1998; Marazziti et al., 1998), but its role there has been largely unexplored. In a single recent study, La Sala et al. (2015) investigated the role of GPR37 in gonad differentiation, where they found that both GPR37 and prosaposin were co-localized in somatic Sertoli cells (SCs) and that deletion of GPR37 affects germ cell spermatogenesis. GPR37 KO mice are nonetheless still fertile, despite having delayed sperm cell development, reduced testis weights and lower sperm counts and sperm motility (La Sala et al., 2015). Gonadal expression of GPR37 is sexually dimorphic at birth and during postnatal gonad development, with elevated levels seen in male gonads only. Thus, the authors carefully mapped the effect of GPR37 deletion on sperm cell development from ages P0 (postnatal day 0) to P29 and found that GPR37 KO mice had altered proportions of cell types in the germ cell population and that there was a significant level of apoptosis in seminiferous tubules, from P21 and persisting into adulthood (La Sala et al., 2015). Thus, the smaller testis size of GPR37 KO mice was attributed to the reduced number of SCs resulting from impaired proliferation/enhanced apoptosis. Mechanistically, GPR37 KO mice were found to have altered levels of androgen receptor and components of the desert hedgehog (Dhh, the testis-specific member of the hedgehog mitogens) signaling pathway during pre-pubertal SC maturation. Specifically, androgen receptor levels were significantly reduced at P10 and P21, relative to wild type littermates, while Dhh, patched-1, Smoothened and Gli1 were all significantly elevated in GPR37 KOs at P10 and P21, but not in adults (La Sala et al., 2015). Prosaposin expression was unaltered by GPR37 deletion and GPR37L1 was not found in the testis at any stage of development or in adulthood (La Sala et al., 2015). Thus, from this comprehensive study of gonad development in GPR37 KO mice, it can be concluded that GPR37 plays an important role in testicular development but is not necessary for male fertility.

### Other Diseases Associated with GPR37

In addition to the physiological and pathophysiological roles already outlined, changes in GPR37 expression have been associated with both cancer and epigenetic regulation. For example, GPR37 mRNA and protein was found to be significantly downregulated in a panel of tissues from patients with hepatocellular carcinoma (Liu et al., 2014). Interestingly, GPR37 expression was inversely proportional to the histopathological grading of the cancer and Kaplan–Meier survival analysis of patients indicated that those with “low” GPR37 expression had markedly reduced survival time (*n* = 37 vs. 20 “high” GPR37 expressers). Examination of GPR37 siRNA in Huh7 cells suggested that changes in GPR37 levels altered cell proliferation and survival (Liu et al., 2014), although this data was less convincing. At the very least, the evidence suggests that Liu et al. (2014) have identified a potential biomarker for hepatocarcinoma severity and survival. In addition to hepatocarcinoma, GPR37 has also been associated with acute myeloid leukemia, where the gene was found to be hypermethylated in patient samples (Toyota et al., 2001). Likewise, GPR37 mRNA and protein was found to be downregulated in a study of the effects of the epigenetic agent, folic acid, on lymphoblastoid cells (Junaid et al., 2011). In the latter two studies, the functional significance of GPR37 dysregulation remains to be established.

## GPR37L1 Physiology

In contrast to the large number of studies into the physiological role of GPR37, only two studies have examined the importance of GPR37L1 to health and disease.

### GPR37L1 and Cerebellar Development

The same group that originally cloned GPR37 (Marazziti et al., 1997) and generated the first GPR37 KO mouse (Marazziti et al., 2004) has also carefully characterized the effect of GPR37L1 on brain development and behavior. GPR37L1 KO mice displayed precocious cerebellum development that was a direct consequence of premature downregulation of granule neuron precursor cell proliferation and concomitant premature development and maturation of Bergmann glia and Purkinje neurons (Marazziti et al., 2013). The effects of GPR37L1 were linked to dysregulation of sonic hedgehog (Shh) signaling, which is known to stimulate proliferation of granule cell precursors and maturation of Bergmann glia. Western blots of various components of the Shh pathway revealed altered expression in GPR37L1 KOs during P5, P10 and P15, key stages of cerebellar development, while GPR37L1 was found to specifically interact with the Shh receptor, patched-1, by co-immunoprecipitation from wild type tissue (Marazziti et al., 2013). Not surprisingly, such marked changes in cerebellar development translated into phenotypic differences in behavioral tests—GPR37L1 KO mice had enhanced motor skills when assessed for rotarod, negative geotaxis, climbing reflex and wire hanging performance (Marazziti et al., 2013). Although GPR37L1 deletion caused marked developmental changes in the cerebellum, the receptor is yet to be linked to the pathogenesis of disease in the central nervous system, either during development or adulthood.

### GPR37L1: A New Target for Blood Pressure Control?

GPR37L1 has been linked to the control of blood pressure in an exciting yet puzzling study of the genetic causes of human heart failure, where the authors performed microarray analysis on explants from 12 patients with heart failure, relative to two reference libraries (Min et al., 2010). A number of different genes were identified as being differentially regulated according to the microarray; these plus an additional set of genes identified by an unreported *in silico* analysis, which included GPR37L1, were then further examined. Specifically, GPR37L1 was listed as being “downregulated” in cardiovascular disease, although no data was presented to support this claim and *p* > 0.005 for the failing vs. non-failing heart comparison of GPR37L1 gene expression (Min et al., 2010). Adenoviruses were generated to enable expression of each of the identified genes in isolated rat neonatal cardiomyocytes and cell growth and viability was assessed, although this was rather perfunctory. For example, “apoptosis” was determined by cell morphology, while cellular hypertrophy was reported as either “increased” or “decreased” for [^3^H]-phenylalanine incorporation experiments. Nevertheless, GPR37L1 was reported to reduce cell viability, cause apoptosis and reduce cellular growth (Min et al., 2010). The most remarkable observation came from the generation of GPR37L1 KO mice, which were reported to have an astonishing 62 mmHg increase in systolic blood pressure when compared to mice that specifically overexpressed GPR37L1 in the heart (no non-transgenic controls were used for the comparison). Furthermore, GPR37L1 KO mice had evidence of significant cardiac hypertrophy as measured by heart weight to body weight ratios (Min et al., 2010), consistent with prolonged hypertension. Unfortunately, the authors did not include any further details that would explain the mechanism of GPR37L1-mediated blood pressure control, although it is possible that the extreme difference in systolic blood pressure could be explained by differing responsiveness to anesthetic (example traces included in the paper show that GPR37L1 KO mice had far higher heart rate than their cardiac-specific overexpressing counterparts, which may artificially multiply the difference between mice). Only one other study has reported the generation of GPR37L1 KO mice, but blood pressure was not reported (Marazziti et al., 2013). Meanwhile, a small genome-wide association study of 424 sudden cardiac death patients with coronary artery disease reported a SNP with *p* < 0.0001 near GPR37L1, however this SNP was not further validated (Arking et al., 2010). Given the methodological concerns of the original paper by Min et al. (2010), it is imperative that further studies explore the role of GPR37L1 in blood pressure homeostasis. If the phenotype can be confirmed, this work suggests that GPR37L1 agonists would be useful additional drugs for the treatment of hypertension.

## Conclusion

Despite their original discovery as endothelin B receptor-related proteins, it is evident that neither GPR37 nor GPR37L1 are receptors for endothelins or endothelin-related ligands. Instead, GPR37 appears to play a role in DA neurotoxicity and has been linked to the development of autosomal-recessive juvenile parkinsonism as well as major depressive and bipolar disorders. Whether GPR37 is itself neuroprotective or neurotoxic remains controversial, however, as the reported ligand for GPR37 and GPR37L1 appears to protect cells from cytotoxic agents while deletion of the receptor achieves the same outcome. Further confounding our understanding of the physiological significance of GPR37 is contradictory behavioral testing results, with some studies suggesting that GPR37 deletion increases anxiety and others show the opposite. Meanwhile, only two studies have investigated the physiological relevance of GPR37L1. Regardless, GPR37 and GPR37L1 remain promising therapeutic targets for the treatment of neurodegeneration and hypertension, respectively, although many additional studies will be necessary to confirm the mechanisms and relevance of these receptors to disease.

### Conflict of Interest Statement

The author declares that the research was conducted in the absence of any commercial or financial relationships that could be construed as a potential conflict of interest.

## References

[B1] Alexander S. P. Benson H. E. Faccenda E. Pawson A. J. Sharman J. L. Spedding M. (2013). The Concise Guide to PHARMACOLOGY 2013/14: G protein-coupled receptors. Br. J. Pharmacol. 170, 1459–1581. 10.1111/bph.12445 24517644 PMC3892287

[B2] Arai H. Hori S. Aramori I. Ohkubo H. Nakanishi S. (1990). Cloning and expression of a cDNA encoding an endothelin receptor. Nature 348, 730–732. 10.1038/348730a0 2175396

[B3] Arking D. E. Reinier K. Post W. Jui J. Hilton G. O’Connor A. (2010). Genome-wide association study identifies GPC5 as a novel genetic locus protective against sudden cardiac arrest. PLoS ONE 5:e9879. 10.1371/journal.pone.0009879 20360844 PMC2845611

[B4] Bakkaloglu B. O”Roak B. J. Louvi A. Gupta A. R. Abelson J. F. Morgan T. M. (2008). Molecular cytogenetic analysis and resequencing of contactin associated protein-like 2 in autism spectrum disorders. Am. J. Hum. Genet. 82, 165–173. 10.1016/j.ajhg.2007.09.017 18179895 PMC2253974

[B5] Bodenmuller H. Schaller H. C. (1981). Conserved amino acid sequence of a neuropeptide, the head activator, from coelenterates to humans. Nature 293, 579–580. 10.1038/293579a0 7290191

[B6] Campana W. M. Hiraiwa M. O’Brien J. S. (1998). Prosaptide activates the MAPK pathway by a G-protein-dependent mechanism essential for enhanced sulfatide synthesis by Schwann cells. FASEB J. 12, 307–314. 9506474 10.1096/fasebj.12.3.307

[B7] Chaudhuri K. R. Healy D. G. Schapira A. H. National Institute for Clinical E. (2006). Non-motor symptoms of Parkinson’s disease: diagnosis and management. Lancet Neurol. 5, 235–245. 10.1016/S1474-4422(06)70373-8 16488379

[B8] Chung K. K. Dawson V. L. Dawson T. M. (2001). The role of the ubiquitin-proteasomal pathway in Parkinson’s disease and other neurodegenerative disorders. Trends Neurosci. 24, S7–S14. 10.1016/S0166-2236(01)00003-0 11881748

[B9] Cruceanu C. Tan P. P. Rogic S. Lopez J. P. Torres-Platas S. G. Gigek C. O. (2015). Transcriptome sequencing of the anterior cingulate in bipolar disorder: dysregulation of G protein-coupled receptors. Am. J. Psychiatry 10.1176/appi.ajp.2015.14101279 [Epub ahead of print]. 26238605

[B10] Davenport A. P. Alexander S. P. Sharman J. L. Pawson A. J. Benson H. E. Monaghan A. E. (2013). International Union of Basic and Clinical Pharmacology. LXXXVIII. G protein-coupled receptor list: recommendations for new pairings with cognate ligands. Pharmacol. Rev. 65, 967–986. 10.1124/pr.112.007179 23686350 PMC3698937

[B11] Donohue P. J. Shapira H. Mantey S. A. Hampton L. L. Jensen R. T. Battey J. F. (1998). A human gene encodes a putative G protein-coupled receptor highly expressed in the central nervous system. Brain Res. Mol. Brain Res. 54, 152–160. 10.1016/S0169-328X(97)00336-7 9526070

[B12] Dunham J. H. Hall R. A. (2009). Enhancement of the surface expression of G protein-coupled receptors. Trends Biotechnol. 27, 541–545. 10.1016/j.tibtech.2009.06.005 19679364 PMC2731006

[B13] Dunham J. H. Meyer R. C. Garcia E. L. Hall R. A. (2009). GPR37 surface expression enhancement via N-terminal truncation or protein–protein interactions. Biochemistry 48, 10286–10297. 10.1021/bi9013775 19799451 PMC2785071

[B14] Dutta P. O’Connell K. E. Ozkan S. B. Sailer A. W. Dev K. K. (2014). The protein interacting with C-kinase (PICK1) interacts with and attenuates parkin-associated endothelial-like (PAEL) receptor-mediated cell death. J. Neurochem. 130, 360–373. 10.1111/jnc.12741 24749734

[B15] Fredriksson R. Lagerström M. C. Lundin L. G. Schiöth H. B. (2003). The G-protein-coupled receptors in the human genome form five main families. Phylogenetic analysis, paralogon groups, and fingerprints. Mol. Pharmacol. 63, 1256–1272. 10.1124/mol.63.6.1256 12761335

[B16] Fujita-Jimbo E. Yu Z. L. Li H. Yamagata T. Mori M. Momoi T. (2012). Mutation in Parkinson disease-associated, G-protein-coupled receptor 37 (GPR37/PaelR) is related to autism spectrum disorder. PLoS ONE 7:e51155. 10.1371/journal.pone.0051155 23251443 PMC3520984

[B17] Gandia J. Fernandez-Duenas V. Morato X. Caltabiano G. Gonzalez-Muniz R. Pardo L. (2013). The Parkinson’s disease-associated GPR37 receptor-mediated cytotoxicity is controlled by its intracellular cysteine-rich domain. J. Neurochem. 125, 362–372. 10.1111/jnc.12196 23398388

[B18] Gandia J. Morato X. Stagljar I. Fernandez-Duenas V. Ciruela F. (2015). Adenosine A2A receptor-mediated control of pilocarpine-induced tremulous jaw movements is Parkinson’s disease-associated GPR37 receptor-dependent. Behav. Brain Res. 288, 103–106. 10.1016/j.bbr.2015.04.001 25862943

[B19] Goldberg M. S. Fleming S. M. Palacino J. J. Cepeda C. Lam H. A. Bhatnagar A. (2003). Parkin-deficient mice exhibit nigrostriatal deficits but not loss of dopaminergic neurons. J. Biol. Chem. 278, 43628–43635. 10.1074/jbc.M308947200 12930822

[B20] Hallett M. (2012). Parkinson’s disease tremor: pathophysiology. Parkinsonism Relat. Disord. 18(Suppl. 1), S85–S86. 10.1016/S1353-8020(11)70027-X 22166464

[B21] Hiraiwa M. Campana W. M. Martin B. M. O’Brien J. S. (1997). Prosaposin receptor: evidence for a G-protein-associated receptor. Biochem. Biophys. Res. Commun. 240, 415–418. 10.1006/bbrc.1997.7673 9388493

[B22] Imai Y. Soda M. Hatakeyama S. Akagi T. Hashikawa T. Nakayama K. I. (2002). CHIP is associated with Parkin, a gene responsible for familial Parkinson’s disease, and enhances its ubiquitin ligase activity. Mol. Cell 10, 55–67. 10.1016/S1097-2765(02)00583-X 12150907

[B23] Imai Y. Soda M. Inoue H. Hattori N. Mizuno Y. Takahashi R. (2001). An unfolded putative transmembrane polypeptide, which can lead to endoplasmic reticulum stress, is a substrate of Parkin. Cell 105, 891–902. 10.1016/S0092-8674(01)00407-X 11439185

[B24] Imai Y. Soda M. Takahashi R. (2000). Parkin suppresses unfolded protein stress-induced cell death through its E3 ubiquitin-protein ligase activity. J. Biol. Chem. 275, 35661–35664. 10.1074/jbc.C000447200 10973942

[B25] Inoue A. Yanagisawa M. Kimura S. Kasuya Y. Miyauchi T. Goto K. (1989). The human endothelin family: three structurally and pharmacologically distinct isopeptides predicted by three separate genes. Proc. Natl. Acad. Sci. U.S.A. 86, 2863–2867. 10.1073/pnas.86.8.2863 2649896 PMC287019

[B26] Itier J. M. Ibanez P. Mena M. A. Abbas N. Cohen-Salmon C. Bohme G. A. (2003). Parkin gene inactivation alters behaviour and dopamine neurotransmission in the mouse. Hum. Mol. Genet. 12, 2277–2291. 10.1093/hmg/ddg239 12915482

[B27] Ito J. Ito M. Nambu H. Fujikawa T. Tanaka K. Iwaasa H. (2009). Anatomical and histological profiling of orphan G-protein-coupled receptor expression in gastrointestinal tract of C57BL/6J mice. Cell Tissue Res. 338, 257–269. 10.1007/s00441-009-0859-x 19763624

[B28] Junaid M. A. Kuizon S. Cardona J. Azher T. Murakami N. Pullarkat R. K. (2011). Folic acid supplementation dysregulates gene expression in lymphoblastoid cells—implications in nutrition. Biochem. Biophys. Res. Commun. 412, 688–692. 10.1016/j.bbrc.2011.08.027 21867686

[B29] Kitada T. Asakawa S. Hattori N. Matsumine H. Yamamura Y. Minoshima S. (1998). Mutations in the parkin gene cause autosomal recessive juvenile parkinsonism. Nature 392, 605–608. 10.1038/33416 9560156

[B30] Kitao Y. Imai Y. Ozawa K. Kataoka A. Ikeda T. Soda M. (2007). Pael receptor induces death of dopaminergic neurons in the substantia nigra via endoplasmic reticulum stress and dopamine toxicity, which is enhanced under condition of parkin inactivation. Hum. Mol. Genet. 16, 50–60. 10.1093/hmg/ddl439 17116640

[B31] Kohan D. E. Rossi N. F. Inscho E. W. Pollock D. M. (2011). Regulation of blood pressure and salt homeostasis by endothelin. Physiol. Rev. 91, 1–77. 10.1152/physrev.00060.2009 21248162 PMC3236687

[B32] Kubota K. Niinuma Y. Kaneko M. Okuma Y. Sugai M. Omura T. (2006). Suppressive effects of 4-phenylbutyrate on the aggregation of Pael receptors and endoplasmic reticulum stress. J. Neurochem. 97, 1259–1268. 10.1111/j.1471-4159.2006.03782.x 16539653

[B33] Lagerstrom M. C. Schioth H. B. (2008). Structural diversity of G protein-coupled receptors and significance for drug discovery. Nat. Rev. Drug Discov. 7, 339–357. 10.1038/nrd2518 18382464

[B34] La Sala G. Marazziti D. Di Pietro C. Golini E. Matteoni R. Tocchini-Valentini G. P. (2015). Modulation of Dhh signaling and altered Sertoli cell function in mice lacking the GPR37-prosaposin receptor. FASEB J. 29, 2059–2069. 10.1096/fj.14-269209 25609427

[B35] Leng N. Gu G. Simerly R. B. Spindel E. R. (1999). Molecular cloning and characterization of two putative G protein-coupled receptors which are highly expressed in the central nervous system. Brain Res. Mol. Brain Res. 69, 73–83. 10.1016/S0169-328X(99)00092-3 10350639

[B36] Li Z. S. Schmauss C. Cuenca A. Ratcliffe E. Gershon M. D. (2006). Physiological modulation of intestinal motility by enteric dopaminergic neurons and the D2 receptor: analysis of dopamine receptor expression, location, development, and function in wild-type and knock-out mice. J. Neurosci. 26, 2798–2807. 10.1523/JNEUROSCI.4720-05.2006 16525059 PMC6675162

[B37] Liu F. Zhu C. Huang X. Cai J. Wang H. Wang X. (2014). A low level of GPR37 is associated with human hepatocellular carcinoma progression and poor patient survival. Pathol. Res. Pract. 210, 885–892. 10.1016/j.prp.2014.07.011 25169131

[B38] Liu J. Wang C. Y. O’Brien J. S. (2001). Prosaptide D5, a retro-inverso 11-mer peptidomimetic, rescued dopaminergic neurons in a model of Parkinson’s disease. FASEB J. 15, 1080–1082. 10.1096/fj.00-0603fje 11292674

[B39] Lockhart P. J. O’Farrell C. A. Farrer M. J. (2004). It’s a double knock-out! The quaking mouse is a spontaneous deletion of parkin and parkin co-regulated gene (PACRG). Mov. Disord. 19, 101–104. 10.1002/mds.20000 14743368

[B40] Lopes J. P. Morato X. Souza C. Pinhal C. Machado N. J. Canas P. M. (2015). The role of Parkinson’s disease-associated receptor GPR37 in the hippocampus: functional interplay with the adenosinergic system. J. Neurochem. 134, 135–146. 10.1111/jnc.13109 25824528

[B41] Lundius E. G. Stroth N. Vukojevic V. Terenius L. Svenningsson P. (2013). Functional GPR37 trafficking protects against toxicity induced by 6-OHDA, MPP+ or rotenone in a catecholaminergic cell line. J. Neurochem. 124, 410–417. 10.1111/jnc.12081 23121049

[B42] Lundius E. G. Vukojevic V. Hertz E. Stroth N. Cederlund A. Hiraiwa M. (2014). GPR37 protein trafficking to the plasma membrane regulated by prosaposin and GM1 gangliosides promotes cell viability. J. Biol. Chem. 289, 4660–4673. 10.1074/jbc.M113.510883 24371137 PMC3931029

[B43] Maguire J. J. Davenport A. P. (2014). Endothelin@25—new agonists, antagonists, inhibitors and emerging research frontiers: IUPHAR Review 12. Br. J. Pharmacol. 171, 5555–5572. 10.1111/bph.12874 25131455 PMC4290702

[B44] Mandillo S. Golini E. Marazziti D. Di Pietro C. Matteoni R. Tocchini-Valentini G. P. (2013). Mice lacking the Parkinson’s related GPR37/PAEL receptor show non-motor behavioral phenotypes: age and gender effect. Genes Brain Behav. 12, 465–477. 10.1111/gbb.12041 23574697

[B45] Marazziti D. Di Pietro C. Golini E. Mandillo S. La Sala G. Matteoni R. (2013). Precocious cerebellum development and improved motor functions in mice lacking the astrocyte cilium-, patched 1-associated Gpr37l1 receptor. Proc. Natl. Acad. Sci. U.S.A. 110, 16486–16491. 10.1073/pnas.1314819110 24062445 PMC3799331

[B46] Marazziti D. Di Pietro C. Golini E. Mandillo S. Matteoni R. Tocchini-Valentini G. P. (2009). Induction of macroautophagy by overexpression of the Parkinson’s disease-associated GPR37 receptor. FASEB J. 23, 1978–1987. 10.1096/fj.08-121210 19218498

[B47] Marazziti D. Di Pietro C. Mandillo S. Golini E. Matteoni R. Tocchini-Valentini G. P. (2011). Absence of the GPR37/PAEL receptor impairs striatal Akt and ERK2 phosphorylation, ΔFosB expression, and conditioned place preference to amphetamine and cocaine. FASEB J. 25, 2071–2081. 10.1096/fj.10-175737 21372109

[B48] Marazziti D. Gallo A. Golini E. Matteoni R. Tocchini-Valentini G. P. (1998). Molecular cloning and chromosomal localization of the mouse Gpr37 gene encoding an orphan G-protein-coupled peptide receptor expressed in brain and testis. Genomics 53, 315–324. 10.1006/geno.1998.5433 9799598

[B49] Marazziti D. Golini E. Gallo A. Lombardi M. S. Matteoni R. Tocchini-Valentini G. P. (1997). Cloning of GPR37, a gene located on chromosome 7 encoding a putative G-protein-coupled peptide receptor, from a human frontal brain EST library. Genomics 45, 68–77. 10.1006/geno.1997.4900 9339362

[B50] Marazziti D. Golini E. Mandillo S. Magrelli A. Witke W. Matteoni R. (2004). Altered dopamine signaling and MPTP resistance in mice lacking the Parkinson’s disease-associated GPR37/parkin-associated endothelin-like receptor. Proc. Natl. Acad. Sci. U.S.A. 101, 10189–10194. 10.1073/pnas.0403661101 15218106 PMC454186

[B51] Marazziti D. Mandillo S. Di Pietro C. Golini E. Matteoni R. Tocchini-Valentini G. P. (2007). GPR37 associates with the dopamine transporter to modulate dopamine uptake and behavioral responses to dopaminergic drugs. Proc. Natl. Acad. Sci. U.S.A. 104, 9846–9851. 10.1073/pnas.0703368104 17519329 PMC1887553

[B52] Mazzuca M. Q. Khalil R. A. (2012). Vascular endothelin receptor type B: structure, function and dysregulation in vascular disease. Biochem. Pharmacol. 84, 147–162. 10.1016/j.bcp.2012.03.020 22484314 PMC3358417

[B53] Meyer R. C. Giddens M. M. Schaefer S. A. Hall R. A. (2013). GPR37 and GPR37L1 are receptors for the neuroprotective and glioprotective factors prosaptide and prosaposin. Proc. Natl. Acad. Sci. U.S.A. 110, 9529–9534. 10.1073/pnas.1219004110 23690594 PMC3677493

[B54] Min K. D. Asakura M. Liao Y. Nakamaru K. Okazaki H. Takahashi T. (2010). Identification of genes related to heart failure using global gene expression profiling of human failing myocardium. Biochem. Biophys. Res. Commun. 393, 55–60. 10.1016/j.bbrc.2010.01.076 20100464

[B55] Misasi R. Sorice M. Garofalo T. Griggi T. Campana W. M. Giammatteo M. (1998). Colocalization and complex formation between prosaposin and monosialoganglioside GM3 in neural cells. J. Neurochem. 71, 2313–2321. 10.1046/j.1471-4159.1998.71062313.x 9832129

[B56] Mistry R. Dowling M. R. Challiss R. A. (2011). [^35^S]GTPγS binding as an index of total G-protein and Gα-subtype-specific activation by GPCRs. Methods Mol. Biol. 746, 263–275. 10.1007/978-1-61779-126-0_14 21607862

[B57] Murakami T. Shoji M. Imai Y. Inoue H. Kawarabayashi T. Matsubara E. (2004). Pael-R is accumulated in Lewy bodies of Parkinson’s disease. Ann. Neurol. 55, 439–442. 10.1002/ana.20064 14991825

[B58] Negre-Pages L. Grandjean H. Lapeyre-Mestre M. Montastruc J. L. Fourrier A. Lepine J. P. (2010). Anxious and depressive symptoms in Parkinson’s disease: the French cross-sectionnal DoPaMiP study. Mov. Disord. 25, 157–166. 10.1002/mds.22760 19950403

[B59] Omura T. Kaneko M. Okuma Y. Orba Y. Nagashima K. Takahashi R. (2006). A ubiquitin ligase HRD1 promotes the degradation of Pael receptor, a substrate of Parkin. J. Neurochem. 99, 1456–1469. 10.1111/j.1471-4159.2006.04155.x 17059562

[B60] Rask-Andersen M. Masuram S. Schioth H. B. (2014). The druggable genome: evaluation of drug targets in clinical trials suggests major shifts in molecular class and indication. Annu. Rev. Pharmacol. Toxicol. 54, 9–26. 10.1146/annurev-pharmtox-011613-135943 24016212

[B61] Rezgaoui M. Susens U. Ignatov A. Gelderblom M. Glassmeier G. Franke I. (2006). The neuropeptide head activator is a high-affinity ligand for the orphan G-protein-coupled receptor GPR37. J. Cell Sci. 119, 542–549. 10.1242/jcs.02766 16443751

[B62] Rubin L. J. Badesch D. B. Barst R. J. Galie N. Black C. M. Keogh A. (2002). Bosentan therapy for pulmonary arterial hypertension. N. Engl. J. Med. 346, 896–903. 10.1056/NEJMoa012212 11907289

[B63] Sakurai T. Yanagisawa M. Takuwa Y. Miyazaki H. Kimura S. Goto K. (1990). Cloning of a cDNA encoding a non-isopeptide-selective subtype of the endothelin receptor. Nature 348, 732–735. 10.1038/348732a0 2175397

[B64] Scott B. Borgman A. Engler H. Johnels B. Aquilonius S. M. (2000). Gender differences in Parkinson’s disease symptom profile. Acta Neurol. Scand. 102, 37–43. 10.1034/j.1600-0404.2000.102001037.x 10893061

[B65] Shimura H. Hattori N. Kubo S. Mizuno Y. Asakawa S. Minoshima S. (2000). Familial Parkinson disease gene product, parkin, is a ubiquitin-protein ligase. Nat. Genet. 25, 302–305. 10.1038/77060 10888878

[B66] Smith N. J. Milligan G. (2010). Allostery at G protein-coupled receptor homo- and heteromers: uncharted pharmacological landscapes. Pharmacol. Rev. 62, 701–725. 10.1124/pr.110.002667 21079041 PMC2993260

[B67] Smith N. J. Ward R. J. Stoddart L. A. Hudson B. D. Kostenis E. Ulven T. (2011). Extracellular loop 2 of the free fatty acid receptor 2 mediates allosterism of a phenylacetamide ago-allosteric modulator. Mol. Pharmacol. 80, 163–173. 10.1124/mol.110.070789 21498659 PMC3127537

[B68] Southern C. Cook J. M. Neetoo-Isseljee Z. Taylor D. L. Kettleborough C. A. Merritt A. (2013). Screening β-arrestin recruitment for the identification of natural ligands for orphan G-protein-coupled receptors. J. Biomol. Screen. 18, 599–609. 10.1177/1087057113475480 23396314

[B69] Tanabe Y. Fujita-Jimbo E. Momoi M. Y. Momoi T. (2015). CASPR2 forms a complex with GPR37 via MUPP1 but not with GPR37(R558Q), an autism spectrum disorder-related mutation. J. Neurochem. 134, 783–793. 10.1111/jnc.13168 25977097

[B70] Tomita H. Ziegler M. E. Kim H. B. Evans S. J. Choudary P. V. Li J. Z. (2013). G protein-linked signaling pathways in bipolar and major depressive disorders. Front. Genet. 4:297. 10.3389/fgene.2013.00297 24391664 PMC3870297

[B71] Toyota M. Kopecky K. J. Toyota M. O. Jair K. W. Willman C. L. Issa J. P. (2001). Methylation profiling in acute myeloid leukemia. Blood 97, 2823–2829. 10.1182/blood.V97.9.2823 11313277

[B72] Valdenaire O. Giller T. Breu V. Ardati A. Schweizer A. Richards J. G. (1998). A new family of orphan G protein-coupled receptors predominantly expressed in the brain. FEBS Lett. 424, 193–196. 10.1016/S0014-5793(98)00170-7 9539149

[B73] Walker J. K. Gainetdinov R. R. Mangel A. W. Caron M. G. Shetzline M. A. (2000). Mice lacking the dopamine transporter display altered regulation of distal colonic motility. Am. J. Physiol. Gastrointest. Liver Physiol. 279, G311–G318. 10915639 10.1152/ajpgi.2000.279.2.G311

[B74] Wang H. Q. Imai Y. Inoue H. Kataoka A. Iita S. Nukina N. (2008). Pael-R transgenic mice crossed with parkin deficient mice displayed progressive and selective catecholaminergic neuronal loss. J. Neurochem. 107, 171–185. 10.1111/j.1471-4159.2008.05607.x 18691389

[B75] Wei C. J. Augusto E. Gomes C. A. Singer P. Wang Y. Boison D. (2014). Regulation of fear responses by striatal and extrastriatal adenosine A2A receptors in forebrain. Biol. Psychiatry 75, 855–863. 10.1016/j.biopsych.2013.05.003 23820821 PMC4058554

[B76] Yanagisawa M. Inoue A. Ishikawa T. Kasuya Y. Kimura S. Kumagaye S. (1988). Primary structure, synthesis, and biological activity of rat endothelin, an endothelium-derived vasoconstrictor peptide. Proc. Natl. Acad. Sci. U.S.A. 85, 6964–6967. 10.1073/pnas.85.18.6964 3045827 PMC282099

[B77] Yang Y. Nishimura I. Imai Y. Takahashi R. Lu B. (2003). Parkin suppresses dopaminergic neuron-selective neurotoxicity induced by Pael-R in *Drosophila*. Neuron 37, 911–924. 10.1016/S0896-6273(03)00143-0 12670421

[B78] Zeng Z. Su K. Kyaw H. Li Y. (1997). A novel endothelin receptor type-B-like gene enriched in the brain. Biochem. Biophys. Res. Commun. 233, 559–567. 10.1006/bbrc.1997.6408 9144577

[B79] Zhang Y. Gao J. Chung K. K. Huang H. Dawson V. L. Dawson T. M. (2000). Parkin functions as an E2-dependent ubiquitin-protein ligase and promotes the degradation of the synaptic vesicle-associated protein, CDCrel-1. Proc. Natl. Acad. Sci. U.S.A. 97, 13354–13359. 10.1073/pnas.240347797 11078524 PMC27228

